# Genetic diversity of *Glossina fuscipes fuscipes* along the shores of Lake Victoria in Tanzania and Kenya: implications for management

**DOI:** 10.1186/s13071-017-2201-x

**Published:** 2017-05-30

**Authors:** Oliver Manangwa, Gamba Nkwengulila, Johnson O. Ouma, Furaha Mramba, Imna Malele, Kirsten Dion, Mark Sistrom, Farrah Khan, Serap Aksoy, Adalgisa Caccone

**Affiliations:** 1 Vector and Vector Borne Disease Institute, P. O. Box 1026, Tanga, Tanzania; 2 Department of Zoology, University of Dar es Salaam, P. O. Box 35064, Dar es Salaam, Tanzania; 3 Africa Technical Research Centre, Vector Health International, P.O. Box 15500, Arusha, Tanzania; 4 Biotechnology Research Institute, Kenya Agricultural and Livestock Research Organization, P.O. Box 362-00902, Kikuyu, Kenya; 5 Tanzania Veterinary Laboratory Agency (TVLA), P. O. Box 9154, Dar es Salaam, Tanzania; 6 Department of Ecology and Evolutionary Biology, Yale University, New Haven, Connecticut USA; 7 School of Natural Sciences, University of California, Merced, CA USA; 8 Department of Molecular, Cellular, and Developmental Biology, Yale University, New Haven, CT USA; 9 Yale School of Public Health, Yale University, 60 College Street, New Haven, CT USA

**Keywords:** *Glossina fuscipes fuscipes*, Tsetse flies, Trypanosomiasis, Microsatellite genetic diversity, Lake Victoria basin

## Abstract

**Background:**

Tsetse flies (Diptera: Glossinidae) are sole vectors for trypanosomiasis, which affect human health and livestock productivity in Africa. Little is known about the genetic diversity of *Glossina fuscipes fuscipes*, which is an important species in Tanzania and Kenya. The main objective of the study was to provide baseline data to determine the genetic variability and divergence of *G. f. fuscipes* in the Lake Victoria basin of Tanzania and Kenya in order to guide future vector control efforts in the region.

**Findings:**

Two hundred and seventy five *G. f. fuscipes* from 8 sites along the shores of Lake Victoria were screened for genetic polymorphisms at 19 microsatellite loci. Samples were collected from two sites in Kenya and six sites in Tanzania. Four of the Tanzanian sites were located in the Rorya district, on the eastern shores of Lake Victoria, while the other two sites were from Ukerewe and Bukoba districts from the southern and western Lake Victoria shores, respectively. Four genetically distinct allopatric clusters were revealed by microsatellite analysis, which sorted the sampling sites according to geography, with sites separated by as little as ~65 km belonging to distinct genetic clusters, while samples located within ~35 km from each other group in the same cluster.

**Conclusion:**

Our results suggest that there is ongoing genetic admixture within sampling sites located ~35 km from each other, while sites located ~65 km apart are genetically isolated from each other. Similar patterns emerged from a parallel study on *G. f. fuscipes* analyzed from the Lake Victoria Uganda shores. From a control perspective these results suggest that for sites within the same genetic cluster, control efforts should be carried out in a coordinated fashion in order to avoid re-invasions. Future work should focus on better quantifying the extent and spatial patterns of the observed genetic discontinuities of the *G. f. fuscipes* populations along the Tanzanian shores. This will aid in their control by providing guidelines on the geographical extent of the area to be treated at the same time.

**Electronic supplementary material:**

The online version of this article (doi:10.1186/s13071-017-2201-x) contains supplementary material, which is available to authorized users.

## Background

Tsetse flies (Diptera: *Glossina*) remain to be insects of economic and medical importance in sub-Saharan Africa. They transmit pathogenic trypanosomes that cause sleeping sickness to humans and nagana to livestock [1, 2]. The occurrence of the disease in humans and livestock has greatly limited the development of agriculture and human health in the region [3]. Estimates by FAO show, Africa loses over 3 million cattle and other domestic livestock due to trypanosomiasis every year [4]. The annual losses, in terms of reduced meat and milk production and in terms of the costs related to treatment and controlling the disease, have been estimated at US $1.2 billion [5]. No vaccine has been developed for the disease to date both for humans and cattle due to the ability of trypanosome parasites to change their surface proteins by antigenic variation [6, 7]. Drugs which are used to treat cattle have been used for long time; as a result drug resistance is increasing rapidly which in the long run will seriously affect the use of these drugs for animal trypanosomiasis control. Similarly, drugs which are used to treat humans are toxic, expensive and difficult to administer at village settings and also have bad side effects [1, 8]. Therefore, tsetse control remains the most effective method of reducing trypanosome infections in animals and human in sub-Saharan countries [6–9].

*Glossina fuscipes fuscipes* is one of the most important tsetse species in the *Palpalis* group, subgenus *Nermohina* Robineau-Desvoidy [10]. In Tanzania the species is widely distributed along the shores of Lake Victoria supported by vegetation growing close to the water [11]. The species is found at the eastern margin in Uganda which extends further east along the shores of Lake Victoria in Western Kenya. The fly is also found in southern Sudan, Chad, the Central African Republic, the Democratic Republic of Congo (DRC) and Angola [12, 13]. Various methods have been employed in tsetse control in different areas in Africa, but the success of these methods varied. Some of these interventions used in the past included bush clearing (destroying vector habitat), elimination of wild animals (reservoir host of the parasites), insecticide ground spraying, live bait technology and the use of baited traps and targets. Despite the fact that tsetse fly densities were temporarily reduced, some of the methods used in the past are not used today because of environment reason and also they are against animal conservation [2, 5, 6]. Sterile Insect Technique (SIT) and Sequential Aerial Technique (SAT) have become promising methods which have been successful used to eradicate tsetse flies in some parts of Africa. SIT was successfully used to eradicate *Glossina austeni* in Unguja Island in Tanzania [2, 14] and SAT has been successfully applied in the Okavango Delta in northern Botswana in the eradication of *Glossina morsitans centralis* [5]. The success of these control interventions depend much on the biogeographical limits of the target tsetse species in such a way that maximum benefit is gained from the natural barriers to reinvasion of the previous controlled areas [5]. One factor that can improve the application of various control methods is knowledge on tsetse population genetics in addition to ecological information [9]. Studying the genetic differentiation between different populations of *G. f. fuscipes* will lead to novel insights into the relationships between genetically distinct populations, including geographical distribution, hybridization and migration patterns. This knowledge can then be used to inform ongoing or planned vector control programs across a target geographical region to identify the most suitable areas to target control to avoid re-infestation of cleared habitats. Studies on the population structure of *G. f. fuscipes* have been carried out in some parts of East Africa, particularly along Lake Victoria shores in Uganda [8, 15–18], and reported high genetic structuring of the species in the area. All authors confirmed the presence of two distinct lineages of *G. f. fuscipes* in Uganda using mtDNA and microsatellites markers except author 18. The isolation of northern and southern *G. f. fuscipes* populations which were thought to occur as a result of fragmentation during extreme drought in East Africa warrants these populations to be treated separately during eradication. Likewise, population study of another tsetse riverine species *G. palpalis gambiensis* in Senegal also indicated the species was sufficiently isolated and currently eradication is underway [9, 19]. However, population structure of *G. p. palpalis* in Burkina Faso and *G. p. gambiensis* in Equatorial Guinea indicated high gene flow compared to other riverine tsetse species [20, 21]. Similarly, population structure study on the same species in Cameroon indicated the species had heterozygote deficit suggesting suppression to be the best option to control the tsetse species in the area [7].

Little is known on the population structure and gene flow of *G. f. fuscipes* in the Lake Victoria basin in Kenya and Tanzania. The information will be useful in the planning of effective regional control of the tsetse species in the Lake Victoria basin (the Lake Victoria basin includes parts of Uganda, Tanzania and Kenya) under Pan African Tsetse and Trypanosomiasis Eradication Campaign (PATTEC) initiative [22]. Lake Victoria basin is one among important regions which have been identified for tsetse eradication by PATTEC. This report describes patterns of genetic differentiation among *G. f. fuscipes* sampled in Tanzania and Kenya and relates them to the recommendations for guiding future vector control efforts in the region.

## Methods

### Study sites and data collection

**Fig. 1:**
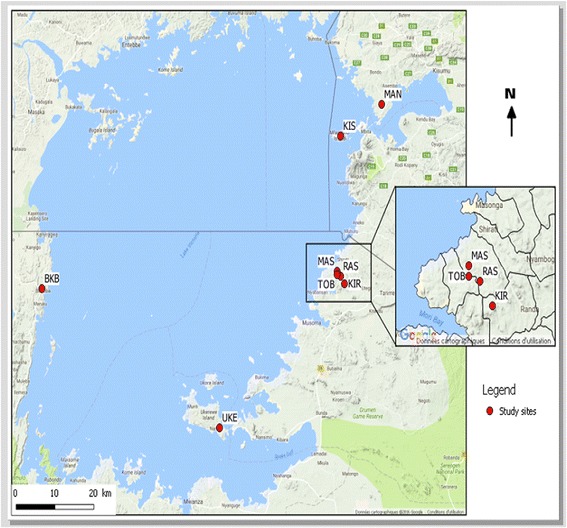
Map showing sampling sites. Location of 8 sampling sites noted by *red dots* and a three letters code to identify sampling sites (BUK, Bukoba; MAN, Manga; KIR, Kirongwe; UKE, Ukerewe; KIS, Kisasi; RAS, Rasi Nyabero; TOB, River Tobwe; MAS, Masonga). The inset shows the location of the four sampling sites in the Rorya district. Lake Victoria is shown in *light blue*, as well as other major lakes in Uganda (Lake Kyoga and Lake Albert). *Lines* depict countries borders and the River Nile

**Fig. 2:**
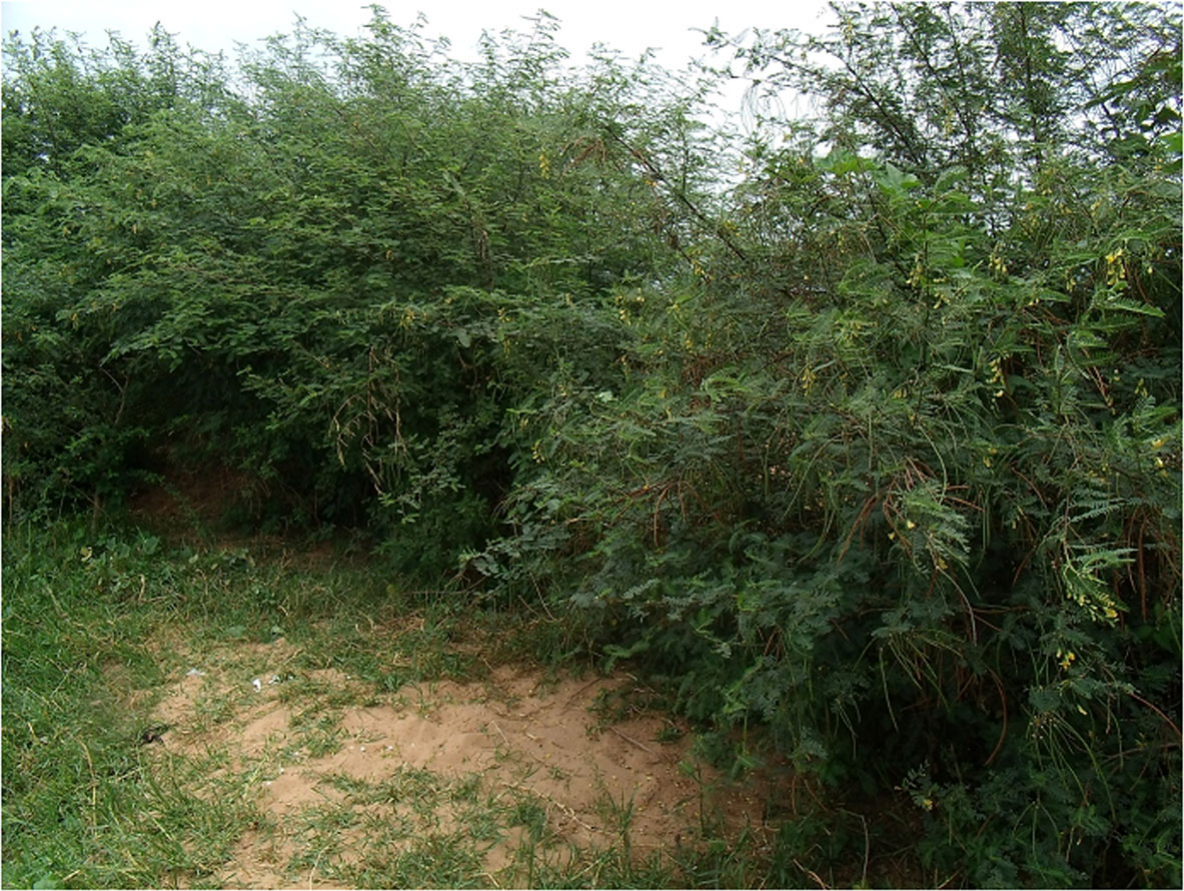
Bushy vegetation in Rasi Nyabero village in Rorya district. A photo showing the vegetation along lake shores in Rorya District in Rasi Nyabero village where flies were trapped. All sites had the same vegetation

*Glossina fuscipes fuscipes* were sampled at 8 localities around Lake Victoria, with 2 sites in Kenya (KIS and MAN) and 6 sites in Tanzania. These sites include 4 locations on the border of Tanzania and Kenya in the Rorya district (MAS, TOB, RAS and KIR), one in the Bukoba district at the border between Uganda and Tanzania (BUK), and one approximately in between these two areas in the Ukerewe district (UKE; Fig. 1). The average distance among the four sampling sites in the Rorya district ranged from 2 to 10 km, the two sampling sites from Kenya (KIS and MAN) are 35 km apart, ~65 km from the Rorya district samples, and ~200 km away from the other two Tanzanian samples from the southern and western shores of Lake Victoria (BUK and UKE), which are also similarly distant from each other (187 km). Overall, 275 individual tsetse flies were collected using biconical and pyramid traps and preserved in cryotubes containing 90% ethanol. Flies from the Tanzanian sites were collected in 2011 while the ones from the two Kenyan sites were collected in 2009. All samples were collected during the dry season. All study sites experience a bimodal rainfall pattern, short rains fall between October and December and long rains from March to May. The average annual rainfall in the basin is estimated to be 1,015 mm [23]. The vegetation of the study sites was bushy vegetation and was the same in all study sites (Fig. 2).

### DNA extraction and PCR analysis

DNA extraction from tsetse legs was conducted using a Qiagen Micro Amp extraction kit (Qiagen, Hilden, Germany) according to the manufacturer’s instructions. PCR and genotyping were conducted for microsatellite markers at 19 previously identified loci that are distributed across the genome [8]. PCR conditions used were in accordance with previously described methods [15]. Microsatellite peaks were scored for each individual sample using GeneMarker software [24]. Table 1 reports the number of flies genetically analyzed for each sampling site.

**Table 1:** Summary information on sampling sites and summary statistics for each samplings site and the patterns of genetic variation at 19 microsatellite loci for *G. f. fuscipes* tsetse flies from eight sampling sites along the Kenyan and Tanzania shores of Lake Victoria

Population	Code	District	Country	Longitude	Latitude	*N*	AR	H_E_	H_O_	F_IS_	Cluster
Bukoba	BUK	Bukoba	Tanzania	34.048944	-1.085306	41	2.89	10.31	7.74	0.35	1
Ukerewe	UKE	Ukerewe	Tanzania	33.141111	-2.059444	51	3.37	11.32	10.32	0.23	2
Rasi Nyabero	RAS	Rorya	Tanzania	31.828056	-1.015	23	3.37	5.52	3.89	0.38	3
Kirongwe	KIR	Rorya	Tanzania	34.077778	-1.0425	24	3	5.07	3.5	0.46	3
Masonga	MAS	Rorya	Tanzania	34.023056	-1.112778	24	3.11	7.44	5.61	0.35	3
River Tobwe	TOB	Rorya	Tanzania	34.023056	-1.094167	24	3.68	6.62	4.42	0.44	3
Kisasi	KIS	Kisasi	Kenya	33.96268	-0.47637	48	4.53	19.85	20.53	-0.02	4
Manga	MAN	Manga	Kenya	34.25125	-0.35534	40	4.94	15.98	16.12	0.02	4

*Abbreviations*: *N*, number of genetically tested samples, *AR* mean allelic richness across all loci; *H*_*O*_, observed heterozygosity, *H*_*E*_ expected heterozygosity, *F*_*IS*_ the inbreeding coefficient

### Genetic analyses

The program Genepop 4.2 [25] was used to test for deviations from Hardy-Weinberg equilibrium, estimate allelic richness (AR), calculate expected and observed heterozygosities (He and Ho), and evaluate levels of inbreeding (Fis). Pairwise F_ST_ values between all sampling sites were calculated in Arlequin v 3.5 [26]. Significance was tested using 10,000 permutations. Genetic differentiation among tsetse sampling sites was analyzed using a model-based Bayesian clustering method implemented in Structure 2.3.3 [27]*.* The optimal number of clusters in the data was calculated using the *ad hoc* statistic ΔK in STRUCUTURE HARVESTER [28]. The STRUCTURE analysis was run for k = 1 to 8, with 10 replicates of each k value, for a million generations and a ‘burn-in’ period lasting the first 200,000 generations. Afterwards, the results from the STRUCTURE analysis were summarized across the 10 replicates using the Greedy method of the software CLUMPP [29], and *distruct* package was used to plot the results. We also used discriminant analysis of principal components (DAPC) [30] conducted with the R *adegenet* package [31] to determine genetic structure. In addition, we used the Bayesian Information Criterion (BIC) to determine the most likely number of clusters in our data [32].

To investigate patterns of migration and identify any probable migrants within our dataset, we used GeneClass2 [33]. GeneClass2 computes the probability of an individual’s observed multilocus genotype belonging to a given population. As such, the program can detect whether an individual is a resident of the sampled population or a first generation migrant. Effective population sizes (N_e_) for the clustered populations were estimated under the linkage disequilibrium model with random mating included in the program NeEstimator v2 [34]. All alleles were taken into account regardless of frequency. Jackknife on loci calculations was implemented to generate 95% confidence bounds.

## Results and discussion

In the present study, a total of 275 tsetse flies were genotyped at 19 microsatellite loci. According to inbreeding coefficient values for each population, no loci deviated significantly from Hardy-Weinberg equilibrium (Table 1, Additional file 1: Table S1). The number of alleles, a measure of genetic diversity, varied from 4.1 in Rasi Nyabero (RAS) to 5.6 in Manga (MAN). The number of alleles per locus varied from 2 in D101 to 15 in GmA06 with an average of 9.4. Mean allelic richness (A_R_) ranged from 2.89 to 4.94 across the sites. No significant differences were observed between observed and expected heterozygosities (H_E_ ranged from 5.07 to 19.85 per site, and H_O_ ranged from 3.50 to 20.53 per site). These values are similar to the ones reported for *G. f. fuscipes* populations around Lake Victoria shores in Uganda [6, 19] and suggest that *G. f. fuscipes* flies in this region harbor a wealth of genetic diversity and thus have the long term potential to adapt to ecological changes in the area.

**Fig. 3:**
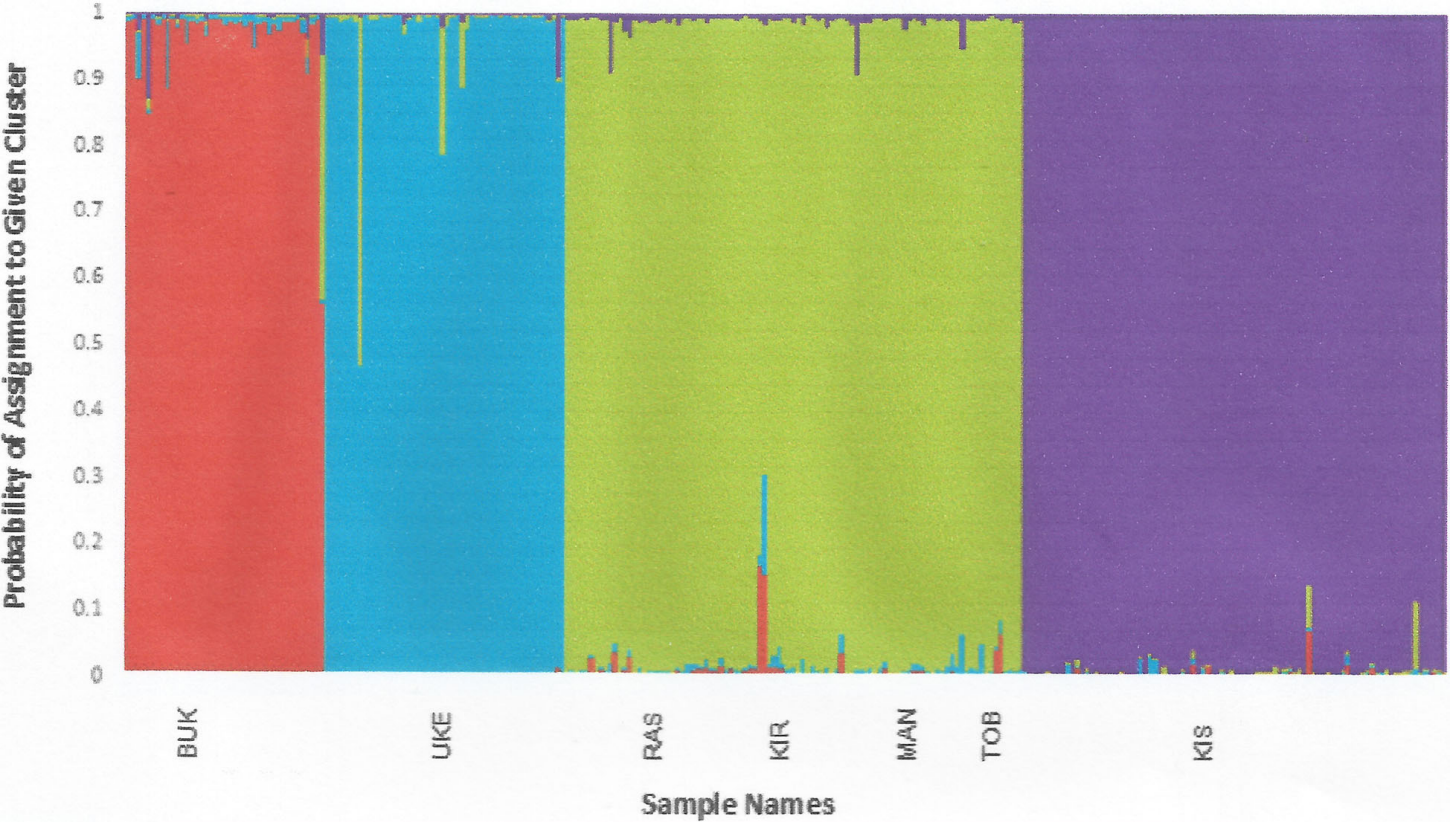
Bayesian clustering obtained using STRUCTURE (27) based on 19 microsatellite loci and 8 sampling sites. Each vertical bar represents the probability of assignment (Y-axis) of one individual (X-axis) to each of the 4 clusters identified by STRUCTURE [27]*.* Different colors represent different genetic clusters: *Red*, Cluster 1 (BUK); *Aqua*, Cluster 2 (UKE); *Green*, Cluster 3 (KIR, RAS, MAS and TOB);* Purple*, Cluster 4 (KIS and MAN). Vertical bars with multiple colors denote admixed individuals, which are assigned to more than one cluster

F_ST_ values revealed significant genetic differentiation among sampling sites over 60–65 km apart, with notable exceptions being the significant F_ST_ values found between two close sampling sites in the Rorya district (KIR and RAS, < 6 km apart, Additional file 1: Table S2) and the lack of significance for F_ST_ values between samples from KIS and either RAS or MAN, located ~ 68–71 km from KIS (Additional file 1: Table S2). When the population structure was investigated using Bayesian clustering and multivariate analyses, the data was most consistent with 4 distinct population clusters (Fig. 3; Additional file 1: Figure S1, and Additional file 1: Figure S2), with F_ST_ values between clusters being all statistically significant and ranging from 0.231 to 0.341 (Additional file 1: Tables S3 and S4). Clusters 1 and 2 group flies from BUK and UK in Bukoba and Ukerewe districts, respectively. Cluster 3 comprises flies from all the Rorya district sampling sites (Rasi Nyabero, RAS; Kirongwe, KIR; Masonga, MAS; and River Tobwe, TOB). Cluster 4 includes the two Kenyan locations (Kisasi, KIS and Manga, MAN). Individual flies collected in sampling sites within these clusters assign mostly to their respective clusters with only a few individuals showing signs of genetic admixture with another cluster. The high genetic differentiation (F_ST_ = 0.341; F_ST_ = 0.308 and F_ST_ = 0.286) recorded in this study is consistent with earlier reports on the same species in other areas of East Africa and is likely a consequence of genetic drift. In the riverine and lacustrine tsetse species, such as *G. f. fuscipes*, wet season dispersion from dry season refugia is thought to establish demes in which genetic drift leads to differentiation [8, 16, 18]. Similar results have been reported also for other riverine tsetse species in West Africa which include *G. tachinoides* in Ghana [35, 36], *G. p. gambiensis* in Niayes-Senegal [9, 37] and *G. p. palpalis* in Cote d’Ivore [38]. However different results of similar species (*G. p. palpalis*) in Cameroon indicated the species formed a large panmixia population suggesting suppression to be the best option for the vector control [7]. In contrast to other riverine tsetse species, *G. swynnertoni*, a savannah species from northern Tanzania, have been reported to have the high level of gene flow [39]. Geographical distance and ecological differences are factors that are involved in these genetic differences of flies [40].

**Fig. 4:**
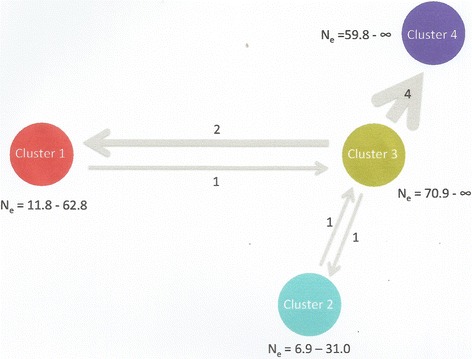
Patterns of migration and effective population size ranges as determined in GeneClass2 and NeEstimator v2, respectively [33, 34]. The *gray* arrows indicate the direction of migration. Line weights are proportional to the number of migrants. The values adjacent to the arrows are the number of migrants identified within our data set. Distances between clusters are not proportional to actual distances between sample sites. Cluster 1: BUK; Cluster 2: UKE; Cluster 3: RAS, KIR, MAS and TOB; Cluster 4: KIS and MAN

The presence of admixed genotypes within clusters 2 and 3 (Fig. 3) suggests ongoing or recent gene flow among sampling locales. This is confirmed by the GeneClass2 analysis aimed to detect first generation migrants, which identified nine likely migrants: three between clusters 1 and 3, two between clusters 2 and 3, and four between clusters 3 and 4 (Fig. 4). However, given the lack of data for intermediate locations, it is not possible to evaluate if this mixing is due to gene flow from intermediate locations or long-range dispersal. These patterns of genetic discontinuities mirror the ones obtained for the same species along the Uganda coast of Lake Victoria, where among similarly spaced samples genetic discontinuities were identified using microsatellite loci [16].

Although estimates of effective population size (Ne) were not uniform among sampling sites, and given the confidence intervals for clusters 3 and 4 (Fig. 4), the Ne estimates may not be very reliable. The high genetic diversity (F_ST_ = 0.341; F_ST_ = 0.308 and F_ST_ = 0.286) recovered at some sampling sites (Table 1) points to large resident populations. Recent reports indicated high and varied *G. f. fuscipes* densities in the sampling sites analyzed in this study [11]. These data indirectly seem to support the existence of local large pockets of tsetse flies at these sampling sites. Further screening of tsetse flies at these and neighboring sites, including temporal collections to capture seasonal variations in fly population sizes and densities, would shed additional light on local tsetse population dynamics, which is of critical relevance for monitoring and control.

## Conclusions

From a control perspective these results suggest that, given the genetic homogeneity among sampling sites from the Rorya district, these sites should be controlled and monitored together to guard against reinvasion from neighboring untreated areas. A similar strategy should be adopted for the region including the two Kenyan sampling sites. However, given that the microsatellite data show that Kenya and the Rorya district samples are genetically distinct even though they are only ~65–80 km apart, control and monitoring effort may not necessarily need to include large geographical areas. Future studies should include a more uniform geographical representation of tsetse infested areas than in the current study to quantify the extent and spatial pattern of the genetic discontinuities found in this study among sampling sites located at varying geographical distances.

## Additional file

Additional file 1: Table S1. Matrix of geographical distances among sampling sites (km). **Table S2.** Genetic differentiation between all population pairs. Values in bold are significant at the 0.05 level. **Table S3.** Genetic summary statistics for all 19 microsatellite loci. Summary statistics are shown for each of the 19 microsatellite loci: AR, allelic richness; H_O_, observed heterozygosity; H_E_, expected heterozygosity; F_IS_, inbreeding coefficient and its *P*-value. **Table S4**. Genetic differentiation between the 4 clusters identified by STRUCTURE. Values in bold are significant at the 0.05 level. *Key*: Cluster 1 = BUK, Cluster 2 = UKE, Cluster 3 = RAS, KIR, MAS, TOB, Cluster 4 = KIS, MAN. **Figure S1**. Delta K Log Likelihood plot for *G. f. fuscipes* clusters using the second order rate of change method [16]. The ΔK plot for a given number of clusters (K) shows that the most likely number of *G. f. fuscipes* clusters from the samples studied is four. **Figure S2.** Bayesian Information Criterion (BIC) *versus* the number of clusters (*k*) generated for discriminant analysis of principal components (DAPC; Jombart et al.*,* [30]) using Adegenet (Jombart, [31]) for all *G. f. fuscipes* microsatellite MLLs. A *k* value of four was chosen to describe the data. **Figure S3.** Discriminant analysis of principal components (DAPC). Assignment of individuals from 8 sampling sites to each of the 4 identified clusters. Points represent individual genotypes sampled from a sampling site and are connected by lines to the 95% confidence ellipse centroid of the respective population. Numbers refer to the clusters identified by the STRUCTURE analyses (Fig. 3) (DOC 328 kb)

## Abbreviations

AR: Allelic richness
BIC: Bayesian Information Criterion
BUK: Bukoba
D101: Primer name derived from loci (D101) for assessing genetic diversity
DAPC: Discriminant Analysis of Principal components
F_IS_: Inbreeding coefficient
GmA06: Primer name derived from the loci of mutant lines of interest for assessing genetic diversity
He: Expected heterozygosity
Ho: Observed heterozygosity
KIR: Kirongwe
KIS: Kisimani
MAN: Manga
MAS: Masonga
Ne: Effective population size
RAS: Rasi Nyabero
UKE: Ukerewe
