# Supplementary material for: Genetic diversity of Glossina fuscipes fuscipes along the shores of Lake Victoria in Tanzania and Kenya: implications for management
Source: Parasit Vectors. 2017 May 30;10:268. doi: 10.1186/s13071-017-2201-x (PMC5450392; doi:10.1186/s13071-017-2201-x)

**Additional file 1**

**Table S1. Matrix of geographical distances among sampling sites (km)**

|  | **KIS** | **MAN** | **BUK** | **UKE** | **KIR** | **RAS** | **MAS** |
| --- | --- | --- | --- | --- | --- | --- | --- |
| **MAN** | 34.79 |  |  |  |  |  |  |
| **BUK** | 244.8 | 279.2 |  |  |  |  |  |
| **UKE** | 198.3 | 226.1 | 186.5 |  |  |  |  |
| **KIR** | 64.24 | 78.81 | 250.1 | 153.7 |  |  |  |
| **RAS** | 68.39 | 84.23 | 247 | 148 | 5.739 |  |  |
| **MAS** | 71.08 | 87.96 | 244.3 | 143.8 | 9.903 | 4.197 |  |
| **TOB** | 69.02 | 85.98 | 244.2 | 145.4 | 8.368 | 3.042 | 2.069 |

**Table S2.** Genetic differentiation between all population pairs. Values in bold are significant at the 0.05 level

|  | BUK | UKE | RAS | KIR | MAS | TOB | KIS |
| --- | --- | --- | --- | --- | --- | --- | --- |
| UKE | **0.341** |  |  |  |  |  |  |
| RAS | **0.200** | **0.263** |  |  |  |  |  |
| KIR | **0.221** | **0.243** | **0.046** |  |  |  |  |
| MAS | **0.238** | **0.286** | 0.012 | **0.051** |  |  |  |
| TOB | **0.237** | **0.308** | -0.012 | **0.029** | -0.004 |  |  |
| KIS | **0.290** | **0.270** | -0.021 | **0.015** | **0.062** | **0.030** |  |
| MAN | **0.269** | **0.278** | 0.004 | **0.035** | **0.085** | **0.043** | **0.023** |

**Table S3.** Genetic summary statistics for all 19 microsatellite loci. Summary statistics are shown for each of the 19 microsatellite loci: AR, allelic richness; H_O_, observed heterozygosity; H_E_, expected heterozygosity; F_IS_, inbreeding coefficient and its *P*-value

|  |  | **AR** | **Ha** | **Ho** | **Fis** | **P-value** |
| --- | --- | --- | --- | --- | --- | --- |
| ***BUK*** | **B05** | 2 | 10.9740 | 13 | -0.1875 | 0.5640 |
| ***BUK*** | **C7b** | 3 | 22.4400 | 18 | 0.2000 | 0.0230 |
| ***BUK*** | **CAG29** | 3 | 3.8861 | 4 | -0.0297 | 1.0000 |
| ***BUK*** | **D05** | 2 | 11.0741 | 3 | 0.7315 | 0.0001 |
| ***BUK*** | **D101** | 3 | 12.3151 | 11 | 0.1081 | 0.6702 |
| ***BUK*** | **GmmA06** | 2 | 7.8571 | 3 | 0.6250 | 0.0162 |
| ***BUK*** | **GmB20** | 3 | 5.7284 | 0 | 1.0000 | 0.0000 |
| ***BUK*** | **GmD15** | 2 | 1.971 | 0 | 1.0000 | 0.0147 |
| ***BUK*** | **GmmH09** | 5 | 10.8987 | 11 | -0.0094 | 0.0879 |
| ***BUK*** | **GmL03** | 1 | 0.0000 | 0 | N/A | N/A |
| ***BUK*** | **GmmL11** | 4 | 11.1967 | 3 | 0.7353 | 0 |
| ***BUK*** | **Gmm8** | 1 | 0.0000 | 0 | N/A | N/A |
| ***BUK*** | **GpB20b** | 6 | 21.5873 | 20 | 0.0746 | 0.3401 |
| ***BUK*** | **C5b** | 1 | 0.0000 | 0 | N/A | N/A |
| ***BUK*** | **GpC10** | 2 | 4.6825 | 3 | 0.3630 | 0.1532 |
| ***BUK*** | **GpCAG133** | 3 | 18.2405 | 9 | 0.5098 | 0.0002 |
| ***BUK*** | **Pgp17** | 5 | 13.5556 | 10 | 0.2647 | 0.204 |
| ***BUK*** | **Pgp28** | 2 | 13.2923 | 8 | 0.4019 | 0.0316 |
| ***BUK*** | **A03b** | 5 | 26.1111 | 31 | -0.1900 | 0.0125 |
| ***UKE*** | **B05** | 5 | 13.6832 | 15 | -0.0973 | 1.0000 |
| ***UKE*** | **C7b** | 2 | 2.9381 | 3 | -0.0213 | 1 |
| ***UKE*** | **CAG29** | 1 | 0.0000 | 0 | N/A | N/A |
| ***UKE*** | **D05** | 2 | 1.9798 | 0 | 1 | 0.0097 |
| ***UKE*** | **D101** | 5 | 4.8791 | 2 | 0.5928 | 0.0003 |
| ***UKE*** | **GmmA06** | 2 | 1.9394 | 2 | -0.0323 | 1.0000 |
| ***UKE*** | **GmB20** | 4 | 4.8763 | 1 | 0.7966 | 0 |
| ***UKE*** | **GmD15** | 4 | 5.8022 | 2 | 0.6578 | 0.0007 |
| ***UKE*** | **GmmH09** | 10 | 33.3030 | 31 | 0.0698 | 0 |
| ***UKE*** | **GmL03** | 1 | 0.0000 | 0 | N/A | N/A |
| ***UKE*** | **GmmL11** | 1 | 0.0000 | 0 | N/A | N/A |
| ***UKE*** | **Gmm8** | 3 | 13.9367 | 11 | 0.2128 | 0.0141 |
| ***UKE*** | **GpB20b** | 6 | 28.3146 | 26 | 0.0826 | 0.0558 |
| ***UKE*** | **C5b** | 2 | 1.0000 | 1 | N/A | N/A |
| ***UKE*** | **GpC10** | 1 | 0.0000 | 0 | N/A | N/A |
| ***UKE*** | **GpCAG133** | 4 | 26.7172 | 20 | 0.2533 | 0.0084 |
| ***UKE*** | **Pgp17** | 4 | 31.7071 | 32 | -0.0093 | 0.2653 |
| ***UKE*** | **Pgp28** | 2 | 19.6923 | 20 | -0.0158 | 1 |
| ***UKE*** | **A03b** | 5 | 24.3069 | 30 | -0.2371 | 0.1837 |
| ***RAS*** | **B05** | 3 | 6.6000 | 4 | 0.4009 | 0.1658 |
| ***RAS*** | **C7b** | 4 | 4.6000 | 3 | 0.3636 | 0.2445 |
| ***RAS*** | **CAG29** | 2 | 1.0000 | 1 | N/A | N/A |
| ***RAS*** | **D05** | 4 | 12.0976 | 10 | 0.177 | 0.188 |
| ***RAS*** | **D101** | 4 | 5.4865 | 2 | 0.6418 | 0.0089 |
| ***RAS*** | **GmmA06** | 3 | 1.6667 | 1 | 0.5 | 0.3366 |
| ***RAS*** | **GmB20** | 4 | 10.9333 | 7 | 0.3649 | 0.0785 |
| ***RAS*** | **GmD15** | 4 | 7.5185 | 8 | -0.0667 | 0.0504 |
| ***RAS*** | **GmmH09** | 6 | 13.4595 | 9 | 0.3374 | 0.0549 |
| ***RAS*** | **GmL03** | 1 | 0.0000 | 0 | N/A | N/A |
| ***RAS*** | **GmmL11** | 2 | 2.9091 | 0 | 1.0000 | 0.0298 |
| ***RAS*** | **Gmm8** | 1 | 0.0000 | 0 | N/A | N/A |
| ***RAS*** | **GpB20b** | 3 | 2.5556 | 1 | 0.6364 | 0.112 |
| ***RAS*** | **C5b** | 1 | 0.0000 | 0 | N/A | N/A |
| ***RAS*** | **GpC10** | 2 | 1.0000 | 1 | N/A | N/A |
| ***RAS*** | **GpCAG133** | 3 | 8.1163 | 5 | 0.3895 | 0.0396 |
| ***RAS*** | **Pgp17** | 6 | 5.6098 | 4 | 0.292 | 0.0257 |
| ***RAS*** | **Pgp28** | 2 | 6.2174 | 5 | 0.2029 | 0.5954 |
| ***RAS*** | **A03b** | 9 | 15.0488 | 13 | 0.1391 | 0.3487 |
| ***KIR*** | **B05** | 4 | 9.2000 | 9 | 0.0224 | 1.0000 |
| ***KIR*** | **C7b** | 4 | 6.7647 | 1 | 0.8596 | 0.0006 |
| ***KIR*** | **CAG29** | 1 | 0.0000 | 0 | N/A | N/A |
| ***KIR*** | **D05** | 4 | 2.9333 | 2 | 0.3231 | 0.0569 |
| ***KIR*** | **D101** | 2 | 7.4286 | 4 | 0.4688 | 0.0774 |
| ***KIR*** | **GmmA06** | 2 | 1.6000 | 0 | 1 | 0.2004 |
| ***KIR*** | **GmB20** | 4 | 12.2093 | 9 | 0.2674 | 0.2059 |
| ***KIR*** | **GmD15** | 3 | 6.1053 | 6 | 0.0182 | 0.5725 |
| ***KIR*** | **GmmH09** | 4 | 8.7600 | 7 | 0.2075 | 0.3 |
| ***KIR*** | **GmL03** | 1 | 0.0000 | 0 | N/A | N/A |
| ***KIR*** | **GmmL11** | 3 | 2.8571 | 0 | 1 | 0.0293 |
| ***KIR*** | **Gmm8** | 2 | N/A | N/A | N/A | N/A |
| ***KIR*** | **GpB20b** | 2 | 4.9091 | 3 | 0.4118 | 0.2004 |
| ***KIR*** | **C5b** | 1 | 0.0000 | 0 | N/A | N/A |
| ***KIR*** | **GpC10** | 2 | 1.6000 | 0 | 1 | 0.2003 |
| ***KIR*** | **GpCAG133** | 2 | 3.6571 | 2 | 0.4603 | 0.1696 |
| ***KIR*** | **Pgp17** | 5 | 7.8718 | 6 | 0.2425 | 0.0709 |
| ***KIR*** | **Pgp28** | 2 | 1.0000 | 1 | N/A | N/A |
| ***KIR*** | **A03b** | 9 | 14.2973 | 13 | 0.093 | 0.0161 |
| ***MAS*** | **B05** | 3 | 9.6744 | 10 | -0.0345 | 0.8153 |
| ***MAS*** | **C7b** | 2 | 6.0870 | 2 | 0.6812 | 0.0283 |
| ***MAS*** | **CAG29** | 1 | 0.0000 | 0 | N/A | N/A |
| ***MAS*** | **D05** | 3 | 12.6222 | 12 | 0.0504 | 0.5862 |
| ***MAS*** | **D101** | 2 | 10.6667 | 8 | 0.2542 | 0.3521 |
| ***MAS*** | **GmmA06** | 4 | 4.6364 | 1 | 0.8000 | 0.0048 |
| ***MAS*** | **GmB20** | 3 | 11.0000 | 8 | 0.2772 | 0.336 |
| ***MAS*** | **GmD15** | 4 | 9.6897 | 7 | 0.2847 | 0.0433 |
| ***MAS*** | **GmmH09** | 5 | 14.7561 | 12 | 0.1906 | 0.04 |
| ***MAS*** | **GmL03** | 2 | 1.9355 | 0 | 1.0000 | 0.031 |
| ***MAS*** | **GmmL11** | 1 | 0.0000 | 0 | N/A | N/A |
| ***MAS*** | **Gmm8** | 3 | 1.6667 | 1 | 0.5 | 0.3337 |
| ***MAS*** | **GpB20b** | 6 | 7.4118 | 7 | 0.0588 | 0.3941 |
| ***MAS*** | **C5b** | 0 | N/A | N/A | N/A | N/A |
| ***MAS*** | **GpC10** | 2 | 1.0000 | 1 | N/A | N/A |
| ***MAS*** | **GpCAG133** | 2 | 11.2000 | 8 | 0.2903 | 0.2109 |
| ***MAS*** | **Pgp17** | 5 | 7.7561 | 4 | 0.4904 | 0.0091 |
| ***MAS*** | **Pgp28** | 3 | 9.9355 | 8 | 0.2 | 0.459 |
| ***MAS*** | **A03b** | 8 | 13.9070 | 12 | 0.1399 | 0.1244 |
| ***TOB*** | **B05** | 4 | 9.1628 | 7 | 0.2403 | 0.1456 |
| ***TOB*** | **C7b** | 3 | 3.4667 | 2 | 0.44 | 0.1431 |
| ***TOB*** | **CAG29** | 1 | 0.0000 | 0 | N/A | N/A |
| ***TOB*** | **D05** | 2 | 10.4186 | 10 | 0.0411 | 1 |
| ***TOB*** | **D101** | 4 | 10.3617 | 7 | 0.3292 | 0.028 |
| ***TOB*** | **GmmA06** | 4 | 5.0000 | 3 | 0.4194 | 0.1206 |
| ***TOB*** | **GmB20** | 3 | 11.2791 | 8 | 0.2956 | 0.1753 |
| ***TOB*** | **GmD15** | 4 | 6.5217 | 6 | 0.0833 | 0.7716 |
| ***TOB*** | **GmmH09** | 6 | 12.3030 | 11 | 0.1089 | 0.369 |
| ***TOB*** | **GmL03** | 1 | 0.0000 | 0 | N/A | N/A |
| ***TOB*** | **GmmL11** | 2 | 2.9091 | 0 | 1 | 0.0313 |
| ***TOB*** | **Gmm8** | 4 | 6.1333 | 0 | 1 | 0.0002 |
| ***TOB*** | **GpB20b** | 6 | 7.2941 | 5 | 0.3277 | 0.1818 |
| ***TOB*** | **C5b** | 1 | 0.0000 | 0 | N/A | N/A |
| ***TOB*** | **GpC10** | 2 | 1.9130 | 0 | 1.0000 | 0.0454 |
| ***TOB*** | **GpCAG133** | 2 | 5.2683 | 2 | 0.6262 | 0.0275 |
| ***TOB*** | **Pgp17** | 7 | 10.8889 | 5 | 0.5464 | 0 |
| ***TOB*** | **Pgp28** | 2 | 5.7619 | 3 | 0.4915 | 0.2225 |
| ***TOB*** | **A03b** | 12 | 17.1489 | 15 | 0.1277 | 0.3431 |
| ***KIS*** | **B05** | 5 | 27.3053 | 28 | -0.0257 | 0.0865 |
| ***KIS*** | **C7b** | 3 | 17.0421 | 16 | 0.0618 | 0.301 |
| ***KIS*** | **CAG29** | 2 | 4.7802 | 5 | -0.0465 | 1 |
| ***KIS*** | **D05** | 3 | 26.8316 | 33 | -0.2329 | 0.0033 |
| ***KIS*** | **D101** | 2 | 5.6842 | 6 | -0.0562 | 1 |
| ***KIS*** | **GmmA06** | N/A | N/A | N/A | N/A | N/A |
| ***KIS*** | **GmB20** | 3 | 11.4842 | 13 | -0.1336 | 1 |
| ***KIS*** | **GmD15** | 5 | 11.8925 | 12 | -0.0091 | 0.3792 |
| ***KIS*** | **GmmH09** | 7 | 37.5158 | 47 | -0.2562 | 0 |
| ***KIS*** | **GmL03** | 3 | 11.4842 | 11 | 0.0426 | 0.6171 |
| ***KIS*** | **GmmL11** | 4 | 12.6344 | 5 | 0.6068 | 0 |
| ***KIS*** | **Gmm8** | 6 | 29.4253 | 28 | 0.0490 | 0.0011 |
| ***KIS*** | **GpB20b** | 6 | 23.5604 | 29 | -0.2340 | 0.1664 |
| ***KIS*** | **C5b** | N/A | N/A | N/A | N/A | N/A |
| ***KIS*** | **GpC10** | 2 | 3.8737 | 4 | -0.0330 | 1 |
| ***KIS*** | **GpCAG133** | 4 | 16.0737 | 19 | -0.1844 | 0.7111 |
| ***KIS*** | **Pgp17** | 7 | 31.2421 | 29 | 0.0725 | 0.6057 |
| ***KIS*** | **Pgp28** | 5 | 33.3579 | 31 | 0.0714 | 0.1083 |
| ***KIS*** | **A03b** | 10 | 33.2316 | 33 | 0.0070 | 0.9473 |
| ***MAN*** | **B05** | 4 | 21.1867 | 21 | 0.0089 | 0.1013 |
| ***MAN*** | **C7b** | 4 | 21.8082 | 21 | 0.0376 | 0.9223 |
| ***MAN*** | **CAG29** | 2 | 2.9155 | 3 | -0.0294 | 1.0000 |
| ***MAN*** | **D05** | 3 | 20.7534 | 23 | -0.1099 | 0.1805 |
| ***MAN*** | **D101** | 2 | 3.8481 | 4 | -0.0400 | 1.0000 |
| ***MAN*** | **GmmA06** | N/A | N/A | N/A | N/A | N/A |
| ***MAN*** | **GmB20** | 6 | 13.1233 | 12 | 0.0867 | 0.0138 |
| ***MAN*** | **GmD15** | 5 | 5.7671 | 6 | -0.0410 | 1.0000 |
| ***MAN*** | **GmmH09** | 6 | 29.1096 | 36 | -0.2408 | 0.0403 |
| ***MAN*** | **GmL03** | 3 | 2.9452 | 3 | -0.0189 | 1.0000 |
| ***MAN*** | **GmmL11** | 4 | 3.9041 | 2 | 0.4912 | 0.0009 |
| ***MAN*** | **Gmm8** | 5 | 23.9565 | 21 | 0.1250 | 0.0012 |
| ***MAN*** | **GpB20b** | 9 | 23.1918 | 25 | -0.0791 | 0.2896 |
| ***MAN*** | **C5b** | N/A | N/A | N/A | N/A | N/A |
| ***MAN*** | **GpC10** | 2 | 3.8356 | 4 | -0.0435 | 1.0000 |
| ***MAN*** | **GpCAG133** | 5 | 15.9178 | 14 | 0.1220 | 0.0014 |
| ***MAN*** | **Pgp17** | 9 | 22.7013 | 20 | 0.1204 | 0.5561 |
| ***MAN*** | **Pgp28** | 5 | 28.7013 | 28 | 0.0247 | 0.0332 |
| ***MAN*** | **A03b** | 10 | 27.9178 | 31 | -0.1121 | 0.2511 |

**Table S4**. Genetic differentiation between the 4 clusters identified by STRUCTURE. Values in bold are significant at the 0.05 level. *Key*: Cluster 1 = BUK, Cluster 2 = UKE, Cluster 3 = RAS, KIR, MAS, TOB, Cluster 4 = KIS, MAN.

|  | Cluster 1 | Cluster 2 | Cluster 3 |
| --- | --- | --- | --- |
| Cluster 2 | **0.341** |  |  |
| Cluster 3 | **0.231** | **0.280** |  |
| Cluster 4 | **0.262** | **0.251** | **0.261** |

**Figure S1**. Delta K Log Likelihood plot for *G. f. fuscipes* clusters using the second order rate of change method [16]. The ΔK plot for a given number of clusters (K) shows that the most likely number of *G. f. fuscipes* clusters from the samples studied is four

**
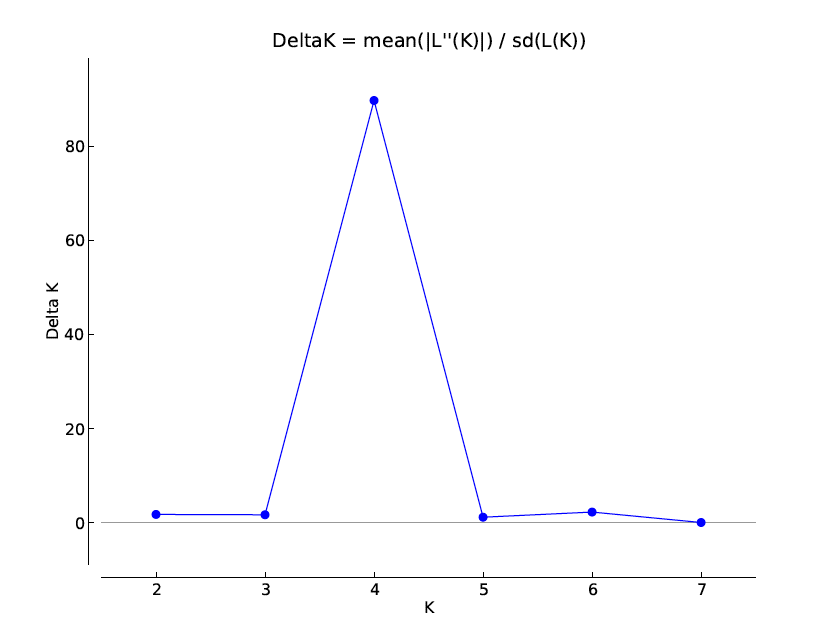
**

**Figure S2.** Bayesian Information Criterion (BIC) *versus* the number of clusters (*k*) generated for discriminant analysis of principal components (DAPC; Jombart et al.*,* 2010) using Adegenet (Jombart, 2008) for all *G. f. fuscipes* microsatellite MLLs. A *k* value of four was chosen to describe the data

**
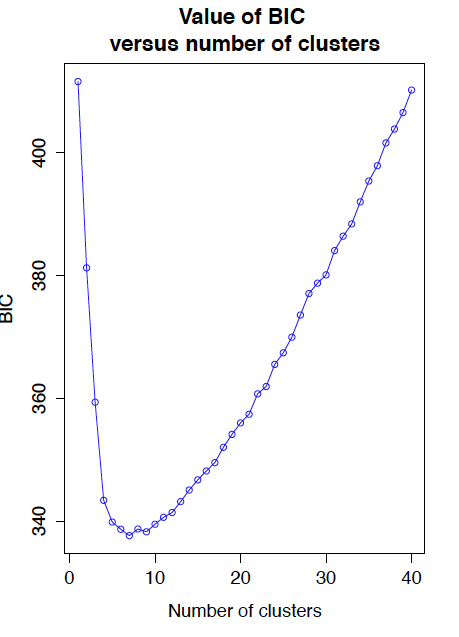
**

**Figure S3.** Discriminant analysis of principal components (DAPC). Assignment of individuals from 8 sampling sites to each of the 4 identified clusters. Points represent individual genotypes sampled from a sampling site and are connected by linesto the 95% confidence ellipse centroid of the respective population. Numbers refer to the clusters identified by the Structure analyses (Fig. 2a)


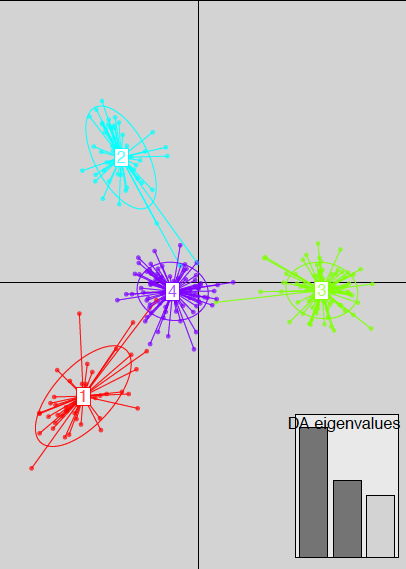

Supplement: Additional file 1: Table S1. — Matrix of geographical distances among sampling sites (km). Table S2. Genetic differentiation between all population pairs. Values in bold are significant at the 0.05 level. Table S3. Genetic summary statistics for all 19 microsatellite loci. Summary statistics are shown for each of the 19 microsatellite loci: AR, allelic richness; HO, observed heterozygosity; HE, expected heterozygosity; FIS, inbreeding coefficient and its P-value. Table S4. Genetic differentiation between the 4 clusters identified by STRUCTURE. Values in bold are significant at the 0.05 level. Key: Cluster 1 = BUK, Cluster 2 = UKE, Cluster 3 = RAS, KIR, MAS, TOB, Cluster 4 = KIS, MAN. Figure S1. Delta K Log Likelihood plot for G. f. fuscipes clusters using the second order rate of change method [16]. The ΔK plot for a given number of clusters (K) shows that the most likely number of G. f. fuscipes clusters from the samples studied is four. Figure S2. Bayesian Information Criterion (BIC) versus the number of clusters (k) generated for discriminant analysis of principal components (DAPC; Jombart et al., [30]) using Adegenet (Jombart, [31]) for all G. f. fuscipes microsatellite MLLs. A k value of four was chosen to describe the data. Figure S3. Discriminant analysis of principal components (DAPC). Assignment of individuals from 8 sampling sites to each of the 4 identified clusters. Points represent individual genotypes sampled from a sampling site and are connected by lines to the 95% confidence ellipse centroid of the respective population. Numbers refer to the clusters identified by the STRUCTURE analyses (Fig. 3) (DOC 328 kb) [file 13071_2017_2201_MOESM1_ESM.doc]
